# Rivaroxaban administration after acute ischemic stroke: The RELAXED study

**DOI:** 10.1371/journal.pone.0212354

**Published:** 2019-02-13

**Authors:** Masahiro Yasaka, Kazuo Minematsu, Kazunori Toyoda, Etsuro Mori, Teruyuki Hirano, Toshimitsu Hamasaki, Hiroshi Yamagami, Takehiko Nagao, Shinichi Yoshimura, Shinichiro Uchiyama, for the RELAXED study group

**Affiliations:** 1 Department of Cerebrovascular Medicine and Neurology, Clinical Research Institute, National Hospital Organization Kyushu Medical Center, Fukuoka, Japan; 2 Departments of Cerebrovascular Medicine and Data Science, National Cerebral and Cardiovascular Center, Suita, Japan; 3 Department of Behavioral Neurology and Neuropsychiatry, United Graduate School of Child Development, Osaka University, Suita, Japan; 4 Department of Stroke and Cerebrovascular Medicine, Kyorin University, Tokyo, Japan; 5 Department of Neurology, Nippon Medical School, Tama-Nagayama Hospital, Tokyo, Japan; 6 Department of Neurosurgery, Hyogo College of Medicine, Hyogo, Japan; 7 International University of Health and Welfare Director, Center for Brain and Cerebral Vessels, Sanno Hospital and Sanno Medical Center, Tokyo, Japan; Indiana University, UNITED STATES

## Abstract

The efficacy of early anticoagulation in acute stroke with nonvalvular atrial fibrillation (NVAF) remains unclear. We performed a study to evaluate the risk of recurrent ischemic stroke (IS) and major bleeding in acute IS patients with NVAF who started rivaroxaban. This observational study evaluated patients with NVAF and acute IS/transient ischemic attack (TIA) in the middle cerebral arterial territory who started rivaroxaban within 30 days after the index IS/TIA. The primary endpoints were recurrent IS and major bleeding within 90 days after the index IS/TIA. The relationship between the endpoints and the time to start rivaroxaban was evaluated by correlation analysis using cerebral infarct volume, determined by diffusion-weighted magnetic resonance images within 48 hours of onset of the index IS/TIA. Of 1309 patients analyzed, recurrent IS occurred in 30 (2.3%) and major bleeding in 11 (0.8%) patients. Among patients with known infarct size (N = 1207), those with small (<4.0 cm^3^), medium (≥4.0 and <22.5 cm^3^), and large (≥22.5 cm^3^) infarcts started rivaroxaban a median of 2.9, 2.9, and 5.8 days, respectively, after the index IS/TIA. Recurrent IS was significantly less frequent when starting rivaroxaban ≤14 days versus ≥15 days after IS (2.0% versus 6.8%, *P* = 0.0034). Incidences of recurrent IS and major bleeding in whom rivaroxaban was started <3 days (N = 584) after IS were also low: 1.5% and 0.7%, respectively. Initiation of rivaroxaban administration in acute IS or TIA was associated with a low recurrence of IS (2.3%), and a low incidence of major bleeding events (0.8%) for 90 days after the index stroke. For the prevention of recurrent attacks in acute IS patients with NVAF, it is feasible to start the administration of rivaroxaban within 14 days of onset. Rivaroxaban started within 3 days of onset may be a feasible treatment option for patients with a small or medium-sized infarction.

## Introduction

The efficacy of early anticoagulation in acute stroke patients with nonvalvular atrial fibrillation (NVAF) has not been established. The recurrence rate of cardioembolic stroke is high during the acute phase (1.6%–10.0% within 7–14 days of onset), which usually worsens prognosis [1,2]. Early anticoagulation after the onset of ischemic stroke (IS) may be useful to prevent recurrence, but this remains to be proven. Anticoagulation with warfarin has disadvantages, including a transient hypercoagulable state at the start of treatment [3] and an increased risk of bleeding complications [4]. Treatment with nonvitamin K antagonist oral anticoagulants (NOACs), including rivaroxaban is associated with a lower incidence of hemorrhagic stroke versus warfarin [5,6].

Unfortunately, large comparative trials of NOACs versus warfarin for stroke prevention in NVAF patients excluded patients in the acute stroke phase. In the ROCKET-AF study, [5] rivaroxaban was associated with significant reductions in intracranial hemorrhage (ICH) versus warfarin. In the J-ROCKET-AF study conducted in Japan, the ICH incidence with rivaroxaban was half that of warfarin [6].

According to current guidelines, the optimal timing to start warfarin or NOACs is within 2 weeks of stroke onset [7,8]. We performed the RELAXED (Recurrent Embolism Lessened by rivaroxaban, an Anti-Xa agent, of Early Dosing for acute ischemic stroke and transient ischemic attack [TIA] with atrial fibrillation [AF]) study to evaluate the risk of recurrent IS and major bleeding associated with rivaroxaban for acute IS or TIA patients with NVAF and investigate relationship between the risk and timing to start rivaroxaban.

## Materials and methods

### Ethics statement

The study protocol and associated documents were reviewed and approved by the Institutional Review Boards of each participating study center in S1 Appendix. This study was conducted in compliance with the Ministry of Health, Labour and Welfare Ethical Guidelines for Clinical Research (MHLW Notification No. 415 [2008]), in addition to the Declaration of Helsinki. All patients (or guardians of participants in the case that patients could not communicate verbally) provided written informed consent.

### Study registrations

The study was registered at ClinicalTrials.gov (NCT02129920) and UMIN-clinical trials registry (UMIN000013932).

### Design

The detailed design and rationale of this study were previously published [9]. The registration and study periods were between February 2014 and April 2016. During the observation period (90 days after onset of index stroke), rivaroxaban was administered according to its approved dosage/administration schedule in Japan [6]. If the creatinine clearance was ≥50 mL/min or 15–49 mL/min, a once-daily dose of 15 mg or 10 mg was planned to be administered, respectively. The cerebral infarct size of the index IS/TIA was measured using diffusion-weighted images (DWI) by magnetic resonance imaging (MRI) performed within 48 hours after the event. An independent MRI Imaging Evaluation Committee evaluated the images.

### Subjects

Patients with NVAF complicated with acute IS or TIA were enrolled consecutively using an internet-based enrollment system if they met the following criteria: patients who were hospitalized or those who visited the hospital as outpatients within 48 hours of the onset of acute IS or TIA; infarct in the middle cerebral artery area demonstrated by DWI or TIA showing symptoms corresponding to this area with negative DWI and disappearing within 24 hours; and receiving treatment with rivaroxaban that started ≤30 days after the onset of acute IS or TIA, regardless of anticoagulation initiated with unfractionated heparin and subsequently substituted by rivaroxaban. TIA was defined as focal neurological symptoms corresponding to the middle cerebral arterial area with negative DWI and disappearing within 24 hours.

Major exclusion criteria were hypersensitivity to rivaroxaban; presence of clinically significant hemorrhage, including gastrointestinal hemorrhage; moderate or severe liver disorder (Child-Pugh class B or C); renal failure (creatinine clearance: <15 mL/min); poorly controlled hypertension (>180/100 mmHg); pregnant women or those likely to become pregnant; treatment with HIV protease inhibitors; treatment with oral or injectable formulation of azole antifungal drugs; acute bacterial endocarditis; anticoagulation initiated with warfarin, dabigatran, apixaban, or edoxaban and then substituted by rivaroxaban; and patients not considered eligible for the study by the investigator.

### Endpoints

#### Primary endpoints

The primary endpoints were recurrence of IS (lasting >24 h, confirmed by MRI/computed tomography); major bleeding, such as symptomatic ICH or hemorrhagic infarction (parenchymal hemorrhage grade 2 and exacerbation of National Institutes of Health Stroke Scale [NIHSS] score ≥4); and other major bleeding according to the criteria defined by the International Society on Thrombosis and Hemostasis [10,11].

#### Secondary endpoints

Secondary endpoints were the incidence of IS and TIA; composite cardiovascular events including IS, TIA, systemic embolism, acute coronary syndrome, deep vein thrombosis, pulmonary embolism, other ischemic diseases, revascularization, cardiovascular death, and total death; any bleeding events; ICH; hemorrhagic transformation of cerebral infarcts; and other adverse events (AEs). AEs were collected and coded in accordance with MedDRA version 19.0. Additionally, we analyzed the incidence of recurrence of IS and occurrence of major hemorrhage according to whether or not heparin was used.

Any hemorrhage and ischemic or other events related to acute revascularization therapy (e.g., recombinant tissue plasminogen activator [rt-PA] or endovascular thrombectomy) were judged by an independent event adjudication committee. These events were not included as endpoints in order to evaluate the risk of IS and major bleeding without influence of acute revascularization.

### Statistical methods

Sample size calculation details were previously reported [9]. A total of 2000 patients were planned to be enrolled. Before the formal analysis, subjects were classified into several groups based on the infarct size and the timing to start rivaroxaban: three groups by tertile range of infarct size, small [<4.0 cm^3^], medium [≥4.0 and <22.5 cm^3^], and large [≥22.5 cm^3^]), and four groups by start date of rivaroxaban, <3 days, 3–7 days, 8–14 days, and ≥15 days from the index IS/TIA. Subject demographic data and the endpoints of recurrent IS and major bleeding were analyzed descriptively. Continuous variables were expressed as means ± SD or medians (interquartile range) and categorical variables were expressed as numbers and percentages. The group comparisons were conducted by chi-square test for categorical variables, and one-way analysis of variance or Kruskal–Wallis test for continuous variables. Cox regression analyses were used to evaluate an effect of the following factors on outcomes: lesion size, time of rivaroxaban initiation, heparin treatment, and other subject baseline characteristics. When fitting Cox regression to the data, Firth’s penalized likelihood approach was used to address issues caused by the small number of events. The base model for the Cox regression analysis included timing to start rivaroxaban administration and infarct size as factors. Factors found to be relevant in the univariate analyses were added to the base model. The relationship between the endpoints and the time to start rivaroxaban treatment during the acute stage of IS was determined by analyzing the correlations between primary endpoints, including recurrent IS or major bleeding and the cerebral infarct size. All tests were two-sided, and a P-value <0.05 was considered significant. All statistical analyses were performed using SAS Version 9.1.3 for Windows (SAS Institute Inc., Cary, NC, USA).

## Results

### Subjects and treatment

**Fig 1:**
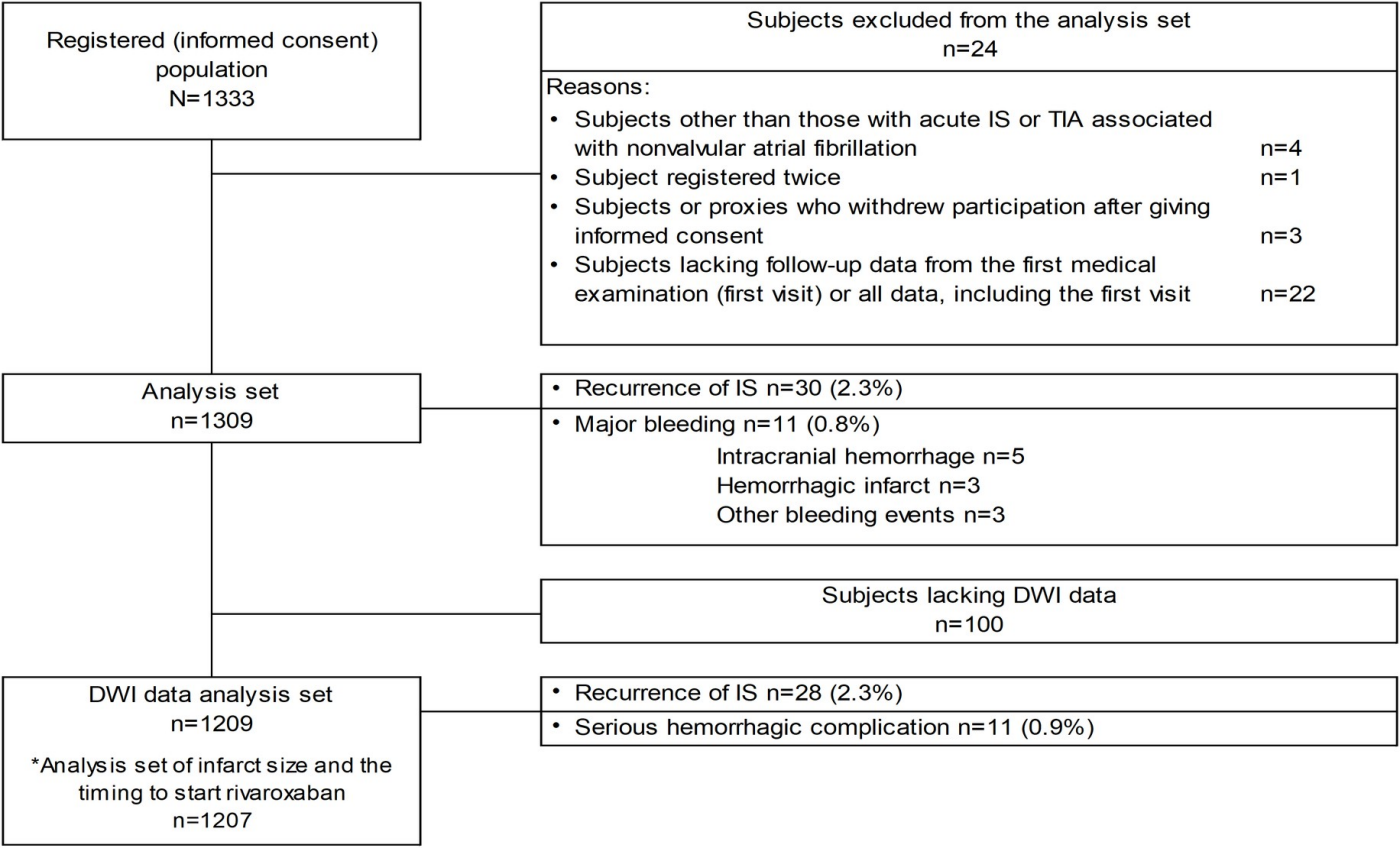
Flow of patients in the study. *Analyses of infarct size and the timing to start rivaroxaban were based on 1207 patients who had both infarct size and administration timing data. DWI, diffusion-weighted images; IS, ischemic stroke; TIA, transient ischemic attack.

In total, 1333 patients were enrolled from 157 sites across Japan. Of these, 1309 patients were included in the analysis. Overall, the majority of patients were male (755 [57.7%]) with a mean age of 77.1 ± 9.6 years (Table 1). Most patients (80.1%) had not received anticoagulants before stroke onset; 97.3% had suffered a stroke, and 2.7% had suffered a TIA. When patients were stratified by cerebral infarct size, the major differences between groups were of the proportion of NIHSS at admission (*P*<0.0001), CHADS_2_ score before onset (*P* = 0.05), and rt-PA treatment or acute endovascular treatment (*P*<0.0001). No significant relationship was found between infarct size and dose. DWI data were available in 1209 patients, which comprised the DWI data analysis set. Analyses of infarct size and the timing to start rivaroxaban were based on 1207 patients who had both infarct size and administration timing data (Fig 1).

**Table 1:** Patient baseline demographic and clinical characteristics.

		Total		Small infarct (<4.0 cm^3^)		Medium infarct (4.0≤&<22.5 cm^3^)		Large infarct (≥22.5 cm^3^)		*P*-value
Gender, n (%)	Male	1308	755 (57.7)	411	228 (55.5)	393	227 (57.8)	404	230 (56.9)	0.802^a^
Age										
Mean years (SD)		1308	77.1 (9.6)	411	77.1 (9.1)	393	77.2 (9.6)	404	77.4 (10.1)	0.943^b^
Age ≥75 years, n (%)			828 (63.3)		263 (64.0)		246 (62.6)		260 (64.4)	0.862^a^
Weight in kg, mean (SD)		1294	57.3 (12.1)	409	57.3 (12.3)	390	57.2 (12.4)	397	56.9 (11.9)	0.859^b^
Heart failure, n (%)	Yes	1198	168 (14.0)	375	53 (14.1)	365	52 (14.2)	369	58 (15.7)	0.794^a^
Hypertension, n (%)	Yes	1286	862 (67.0)	410	283 (69.0)	388	270 (69.6)	394	253 (64.2)	0.208^a^
Diabetes, n (%)	Yes	1296	212 (16.4)	407	78 (19.2)	392	68 (17.3)	400	53 (13.3)	0.069^a^
CLcr mL/min, median (Q1–Q3)		1291	58.8 (45.9–75.4)	409	58.6 (46.5–73.4)	389	58.0 (44.9–76.4)	395	60.1 (46.1–77.9)	0.473^c^
Anticoagulants before onset, n (%)	Yes	1308	260 (19.9)	411	87 (21.2)	393	75 (19.1)	404	81 (20.0)	0.762^a^
Antiplatelet medicine before onset, n (%)	Yes	1308	277 (21.2)	411	90 (21.9)	393	84 (21.4)	404	83 (20.5)	0.893^a^
Stroke/TIA, n (%)	Stroke	1308	1273 (97.3)	401	401 (100.0)	393	393	404	404	-
	TIA		35 (2.7)	10	10 (100.0)		-		-	
NIHSS at admission, median(Q1–Q3)		1301	8 (3–17)	411	3 (1–7)	391	9 (4–16)	400	16 (9–22)	<0.0001^c^
CHADS_2_ before onset, median (Q1–Q3)		1308	2 (1–2)	411	2 (1–3)	393	2 (1–3)	404	2 (1–2)	0.050^c^
HAS-BLED before onset, median (Q1–Q3)		1308	2 (1–2)	411	2 (1–2)	393	2 (1–2)	404	2 (1–2)	0.449^c^
rt-PA treatment or acute endovascular treatment, n (%)	Yes	1308	424 (32.4)	411	104 (25.3)	393	159 (40.5)	404	132 (32.7)	<0.0001^a^
Heparin administration, n (%)	Yes	1308	638 (48.8)	411	210 (51.1)	393	198 (50.4)	404	189 (46.8)	0.421^a^
CLcr (mL/min), n (%)	<50	1291	414 (32.1)	409	130 (31.8)	389	138 (35.5)	395	119 (30.1)	0.262^a^
Rivaroxaban dose	10 mg	1305	440 (33.7)	410	136 (33.2)	393	140 (35.6)	404	136 (33.7)	0.742^a^

^a^, χ^2^ test

^b^, Analysis of variance

^c^, Kruskal–Wallis test

Abbreviations: CLcr, creatinine clearance; NIHSS, National Institute of Health Stroke Scale; rt-PA, recombinant tissue plasminogen activator; SD, standard deviation; TIA, transient ischemic attack.

### Primary endpoints

**Fig 2:**
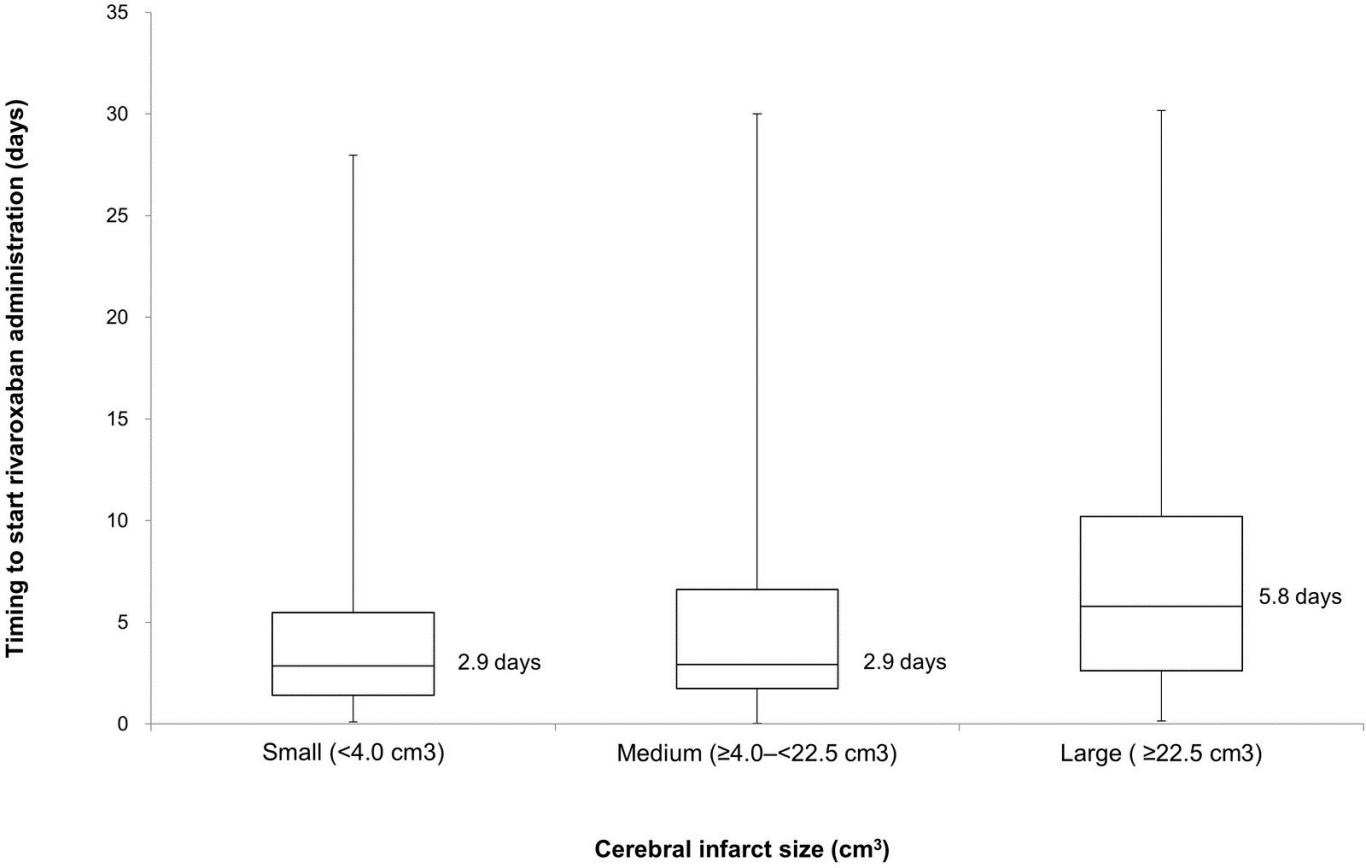
Correlation between timing to start rivaroxaban administration and cerebral infarct size.

**Fig 3:**
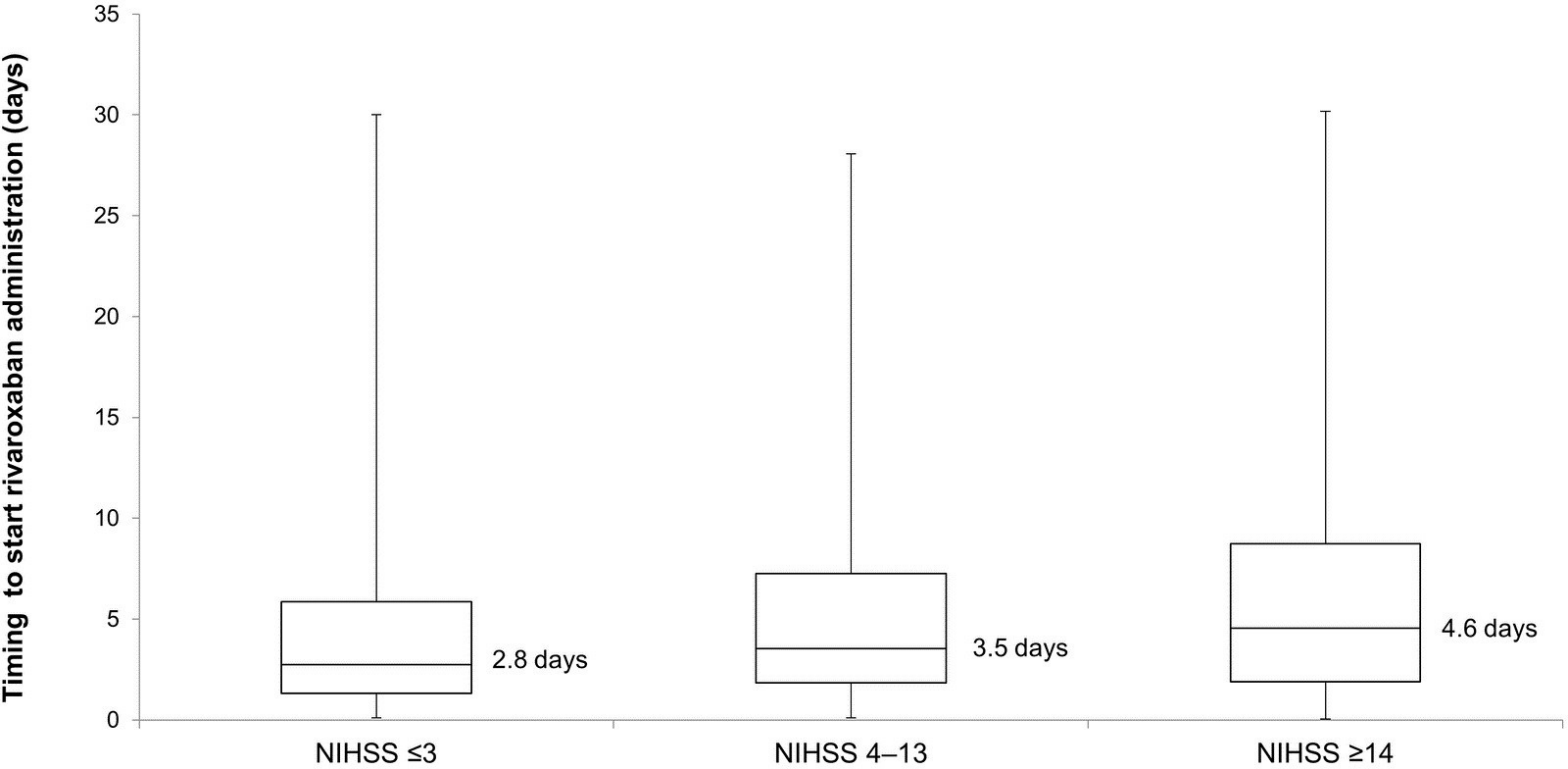
Correlation between timing to start rivaroxaban administration and NIHSS. NIHSS, National Institutes of Health Stroke Scale.

Recurrence of IS occurred in 30 (2.3%) patients and major bleeding in 11 (0.8%) of 1309 patients. Major bleeding events were ICH in five patients; hemorrhagic infarction in three patients; and other bleeding events in three patients (gastrointestinal bleeding). In the patients with known infarct size and known timing to start rivaroxaban (n = 1207), rivaroxaban was used at the early stage for small infarcts (median = 2.9 days, interquartile range [IQR] = 1.4–5.5) and medium infarcts (median = 2.9 days, IQR = 1.8–6.6) and at a later stage for large infarcts (median = 5.8 days, IQR = 2.6–10.2) (Fig 2). Regarding the correlation between the NIHSS scores and the timing to start rivaroxaban administration (Fig 3), patients who had lower NIHSS scores underwent earlier administration of rivaroxaban. The multivariate analysis demonstrated that recurrent IS was associated with the start of rivaroxaban administration ≥15 days after onset (*P* = 0.0021), and major bleeding was associated with use of antiplatelet therapy before onset (*P* = 0.0369) (Tables 2 and 3). In five of the 11 patients with major bleeding, antiplatelet therapy (aspirin in three and clopidogrel in two patients) was administered before onset.

**Table 2:** Univariate and multivariate analysis of the associations between Ischemic stroke recurrence and lesion size, timing to start rivaroxaban administration, and other background characteristics (using the Firth method).

		Number of pts with event (%)		Odds ratio (test/reference)	
Model	Factor	Test	Reference	(95% CI)	P-value
Univariate analysis	Sex	Female 9/553 (1.6%)	Male 21/754 (2.8%)	0.595 (0.275–1.288)	0.188
	Age	≥75 years 20/827 (2.4%)	<75 years 10/480 (2.1%)	1.138 (0.536–2.416)	0.737
	Weight (continuous, per 10)	-	-	0.973 (0.723–1.310)	0.856
	Heart failure	Present 3/94 (3.2%)	Absent 27/1213 (2.2%)	1.65 (0.529–5.149)	0.388
	Hypertension	Present 24/861 (2.8%)	Absent 6/446 (1.3%)	1.983 (0.828–4.748)	0.125
	Diabetes	Present 7/212 (3.3%)	Absent 23/1095 (2.1%)	1.666 (0.721–3.847)	0.232
	CLcr (mL/min)	<50 11/414 (2.7%)	≥50 19/893 (2.1%)	1.278 (0.611–2.676)	0.515
	Heparin	Present 19/636 (3.0%)	Absent 11/671 (1.6%)	1.814 (0.868–3.792)	0.114
	Anticoagulants before onset	Present 8/260 (3.1%)	Absent 22/1047 (2.1%)	1.534 (0.688–3.422)	0.296
	Antiplatelet medicine before onset	Present 10/277 (3.6%)	Absent 20/1030 (1.9%)	1.935 (0.908–4.125)	0.087
	Antiplatelet therapy after onset	Present 7/189 (3.7%)	Absent 23/1118 (2.1%)	1.916 (0.828–4.431)	0.129
	NIHSS at admission (continuous per 1)	-	-	1.002	0.937
Multivariate analysis	Timing to start rivaroxaban administration	≥15 days 6/79 (7.6%)	≤14 days 22/1128 (2.0%)	4.465 (1.754–11.367)	0.0017
(base model)	Infarct size	>22.4 cm^3^ 10/404 (2.5%)	≤22.4 cm^3^ 18/803 (2.2%)	0.917 (0.415–2.026)	0.8296
Multivariate analysis	Timing to start rivaroxaban administration	≥15 days 6/79 (7.6%)	≤14 days 22/1128 (2.0%)	4.272 (1.69–10.8)	0.0021
(base model +	Infarct size	>22.4 cm^3^ 10/404 (2.5%)	≤22.4 cm^3^ 18/803 (2.2%)	0.944 (0.431–2.07)	0.8856
antiplatelet medicine before onset)	Antiplatelet medicine before onset	Present 9/257 (3.5%)	Absent 19/950 (2.0%)	1.738 (0.792–3.814)	0.1679

Abbreviations: IS, ischemic stroke; CLcr, creatinine clearance; NIHSS, National Institute of Health Stroke Scale.

**Table 3:** Univariate and multivariate analysis of the associations between major bleeding and lesion size, timing to start rivaroxaban administration, and other background characteristics (using the Firth method).

		Number of pts with event (%)		Odds ratio (test/reference)	
Model	Factor	Test	Reference	(95% CI)	P-value
Univariate analysis	Sex	Female 6/553 (1.1%)	Male 5/754 (0.7%)	1.618 (0.517–5.068)	0.409
	Age	≥75 years 8/827 (1.0%)	<75 years 3/480 (0.6%)	1.415 (0.405–4.943)	0.587
	Weight (continuous, per 10)	-	-	0.906 (0.546–1.503)	0.703
	Heart failure	Present 2/94 (2.1%)	Absent 9/1213 (0.7%)	3.427 (0.832–14.109)	0.088
	Hypertension	Present 6/861 (0.7%)	Absent 5/446 (1.1%)	0.61 (0.195–1.911)	0.396
	Diabetes	Present 2/212 (0.9%)	Absent 9/1095 (0.8%)	1.359 (0.334–5.530)	0.669
	CLcr (mL/min)	<50 3/414 (0.7%)	≥50 8/893 (0.9%)	0.886 (0.253–3.097)	0.850
	Heparin	Present 7/636 (1.1%)	Absent 4/671 (0.6%)	1.767 (0.546–5.721)	0.342
	Anticoagulants before onset	Present 2/260 (0.8%)	Absent 9/1047 (0.9%)	1.057 (0.260–4.296)	0.938
	Antiplatelet medicine before onset	Present 5/277 (1.8%)	Absent 6/1030 (0.6%)	3.181 (0.260–4.296)	0.048
	Antiplatelet therapy after onset	Present 1/189 (0.5%)	Absent 10/1118 (0.9%)	0.840 (0.150–4.700)	0.843
	NIHSS at admission (continuous per 1)	-	-	1.066	0.040
Multivariate analysis	Timing to start rivaroxaban administration	≥15 days 0/79 (0.0%)	≤14 days 11/1128 (1.0%)	0.502 (0.029–8.550)	0.6337
(base model)	Infarct size	>22.4 cm^3^ 5/404 (1.2%)	≤22.4 cm^3^ 6/803 (0.7%)	1.847 (0.599–5.697)	0.2860
Multivariate analysis	Timing to start rivaroxaban administration	≥15 days 0/79 (0.0%)	≤14 days 11/1128 (1.0%)	0.526 (0.032–8.66)	0.6533
(base model +	Infarct size	>22.4 cm^3^ 5/404 (1.2%)	≤22.4 cm^3^ 6/803 (0.7%)	1.823 (0.602–5.517)	0.2881
heart failure)	Heart failure	Present 2/90 (2.2%)	Absent 9/1117 (0.8%)	3.166 (0.801–12.514)	0.1004
Multivariate analysis	Timing to start rivaroxaban administration	≥15 days 0/79 (0.0%)	≤14 days 11/1128 (1.0%)	0.48 (0.03–7.73)	0.6047
(base model +	Infarct size	>22.4 cm^3^ 5/404 (1.2%)	≤22.4 cm^3^ 6/803 (0.7%)	1.852 (0.612–5.6)	0.2751
antiplatelet medicine before onset)	Antiplatelet medicine before onset	Present 5/257 (1.9%)	Absent 6/950 (0.6%)	3.239 (1.074–9.766)	0.0369
Multivariate analysis	Timing to start rivaroxaban administration	≥15 days 0/79 (0.0%)	≤14 days 11/1128 (1.0%)	0.385 (0.023–6.397)	0.5059
(base model +	Infarct size	>22.4 cm^3^ 5/404 (1.2%)	≤22.4 cm^3^ 6/803 (0.7%)	1.13 (0.337–3.793)	0.8434
NIHSS at admission)	NIHSS at admission (continuous per 1)	-	-	1.068 (1–1.14)	0.0513

Abbreviations: IS, ischemic stroke; CLcr, creatinine clearance; NIHSS, National Institute of Health Stroke Scale.

### Secondary endpoints

When comparing the timing to start rivaroxaban administration (<3 days, 3–7 days, 8–14 days, and ≥15 days) in 1309 patients, regardless of the infarct size, there was a significant difference among the four groups in the composite of IS and TIA (*P* = 0.0087); composite cardiovascular events (i.e., ischemic stroke, TIA, systemic embolism, acute coronary syndrome, deep vein thrombosis, pulmonary embolism, other ischemic disease, revascularization, cardiovascular death, and total death) (*P* = 0.0042); any bleeding event (*P*<0.0001); and hemorrhagic transformation of cerebral infarcts (*P*<0.0001) (Table 4). The incidences of these events were higher in patients starting rivaroxaban ≥15 days after index IS/TIA than in those starting within 14 days (<3 days, 3–7 days or 8–14 days). Primary and secondary endpoint measures compared by timing to start rivaroxaban administration and size of infarct was described in the S1 Table.

**Table 4:** Primary and secondary endpoint measures compared by timing to start rivaroxaban administration.

	Timing to start rivaroxaban administration					
	<3 days	3–7 days	8–14 days	≥15 days	Unknown	
Total, 1,309	584	435	198	88	4	
Event	n (%)	n (%)	n (%)	n (%)		*X*^*2*^-test *P*-value
Recurrent IS	9 (1.5)	11 (2.5)	4 (2.0)	6 (6.8)	0	0.0216
Major bleeding	4 (0.7)	5 (1.1)	2 (1.0)	0	0	0.6874
Composite of IS and TIA	10 (1.7)	13 (3.0)	5 (2.5)	7 (8.0)	0	0.0087
Composite cardiovascular events	21 (3.6)]	21 (4.8)	10 (5.1)	11 (12.5)	0	0.0042
Any bleeding event	62 (10.6)	82 (18.9)	41 (20.7)	36 (40.9)	0	<0.0001
Intracranial hemorrhage	1 (0.2)	4 (0.9)	0	0	0	0.1670
Hemorrhagic infarction	60 (10.3)	77 (17.7)	41 (20.7)	36 (40.9)	0	<0.0001
Adverse events	27 (4.6)	28 (6.4)	11 (5.6)	6 (6.8)	0	0.5962

Abbreviations: IS, ischemic stroke; TIA, transient ischemic attack.

**Fig 4:**
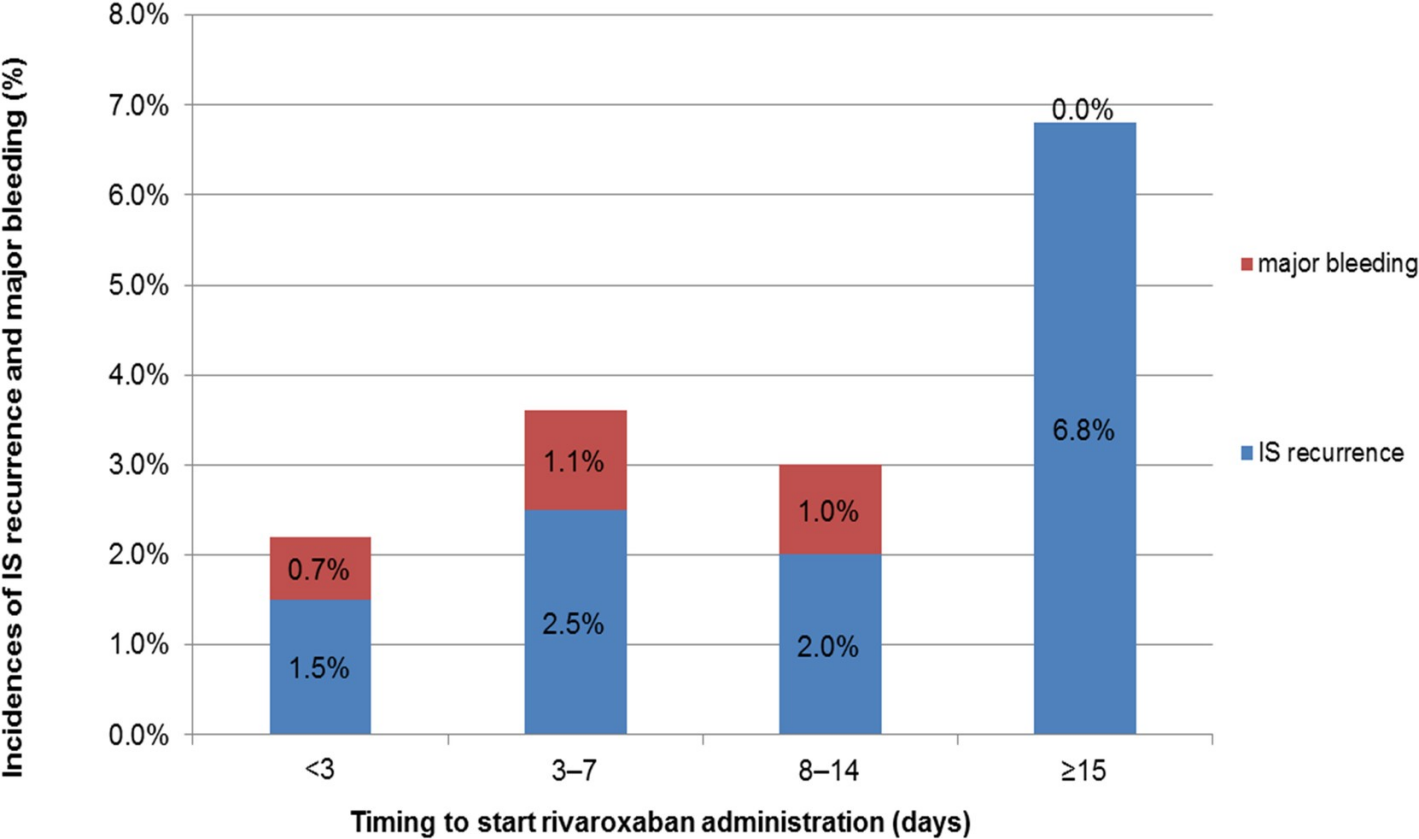
Incidences of ischemic stroke recurrence and major bleeding by timing to start rivaroxaban. IS, ischemic stroke.

**Fig 5:**
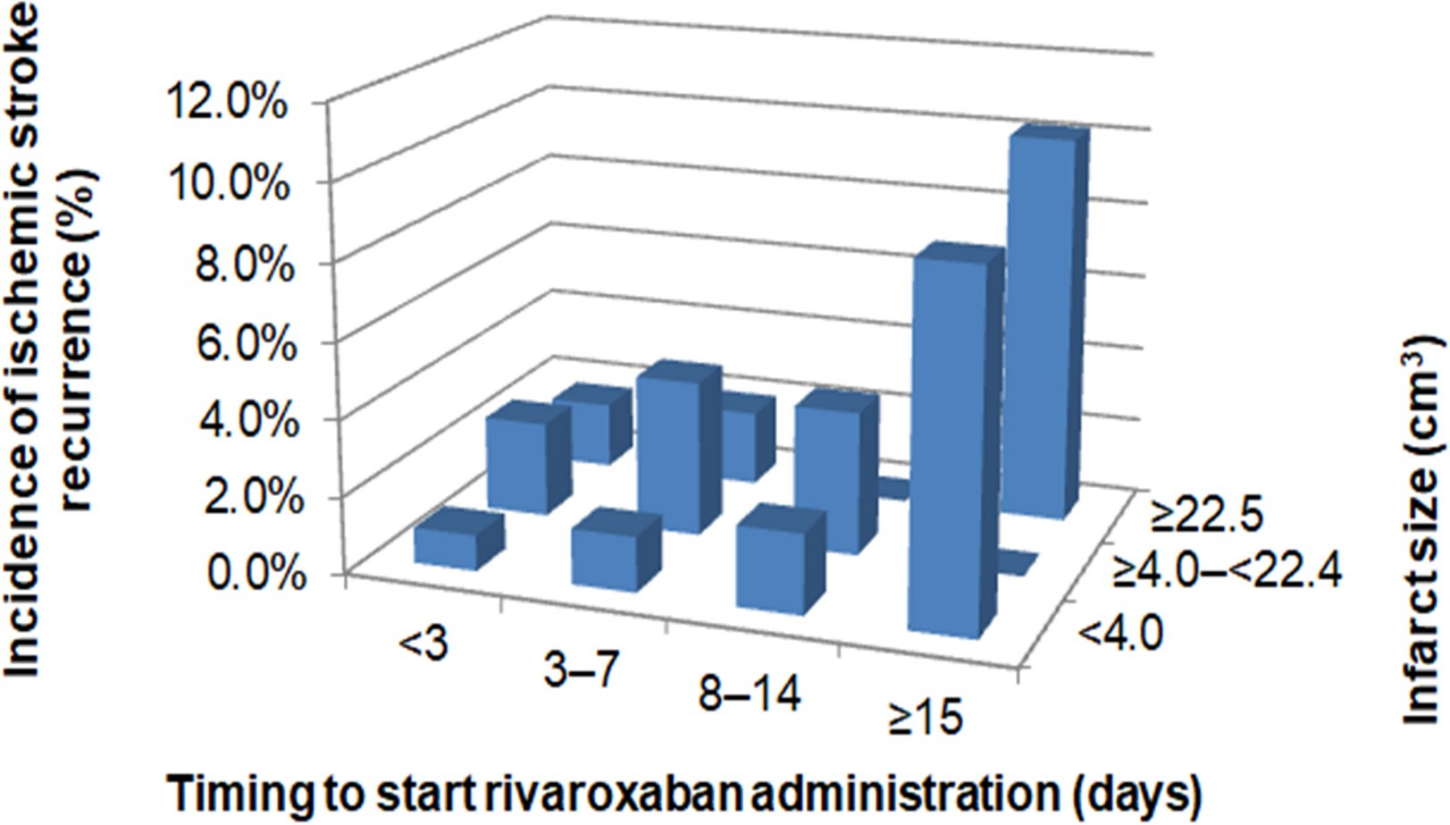
Incidences of ischemic stroke recurrence by timing to start rivaroxaban and cerebral infarct size.

**Fig 6:**
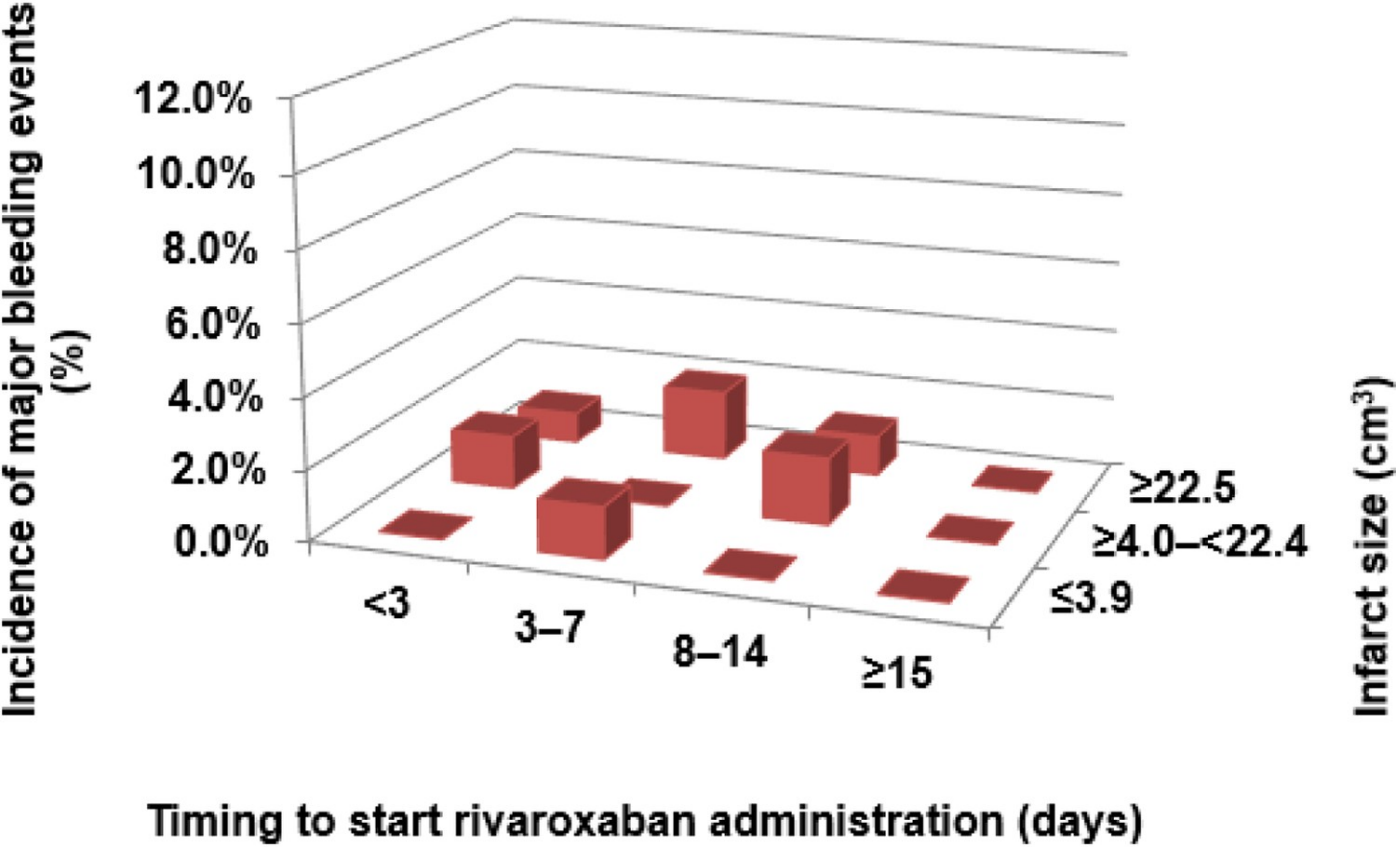
Incidences of major bleeding by timing to start rivaroxaban and cerebral infarct size.

The recurrence rate of ischemic stroke was higher ≥15 days after stroke onset versus ≤14 days of stroke onset (6.8% versus 2.0%, *P* = 0.0034, chi-square test) although there was no significant difference in the incidence of major bleeding within 14 days of stroke onset and that of ≥15 days after stroke onset (0.9% versus 0%, *P* = 0.3704, chi-square test) (Fig 4). Recurrent IS occurred more frequently than major bleeding (2.3% and 0.8%, respectively. *P* = 0.0028, chi-square test). Fig 5 shows the incidence of IS recurrence by infarct size according to the timing to start rivaroxaban administration. The trend of the incidence of IS in each infarct size group was similar to the overall trend. Regarding hemorrhagic complications by cerebral infarct size (Fig 6), they occurred less frequently within 14 days after stroke onset versus ischemic events, but these did not seem to be related to infarct size.

Regarding rt-PA and timing to start rivaroxaban administration, the frequency of rt-PA treatment was 171/584, 29.3% (<3 days), 106/435, 24.5% (3–7 days), 50/198, 25.3% (8–14 days) and 12/88, 13.6% (≥15 days). The frequency of the rt-PA treatment was lower ≥15 days after stroke onset versus ≤14 days of stroke onset (13.6% versus 26.9%, *P* = 0.006, chi-square test).

### Adverse events

The incidence of AEs (except major bleeding) after starting rivaroxaban was summarized in S2 Table. The total incidence of AEs was 4.6% (n = 27) in the group receiving rivaroxaban within <3 days of stroke onset, 6.4% (n = 28) in the group receiving rivaroxaban 3–7 days after stroke onset, 5.6% (n = 11) in the group receiving rivaroxaban 8–14 days after stroke onset, and 6.8% (n = 6) in the group receiving rivaroxaban ≥15 days after stroke onset. There were no significant differences among groups regarding the total incidence of AEs.

#### Incidence of recurrent IS and major bleeding among patients previously treated with heparin

**Fig 7:**
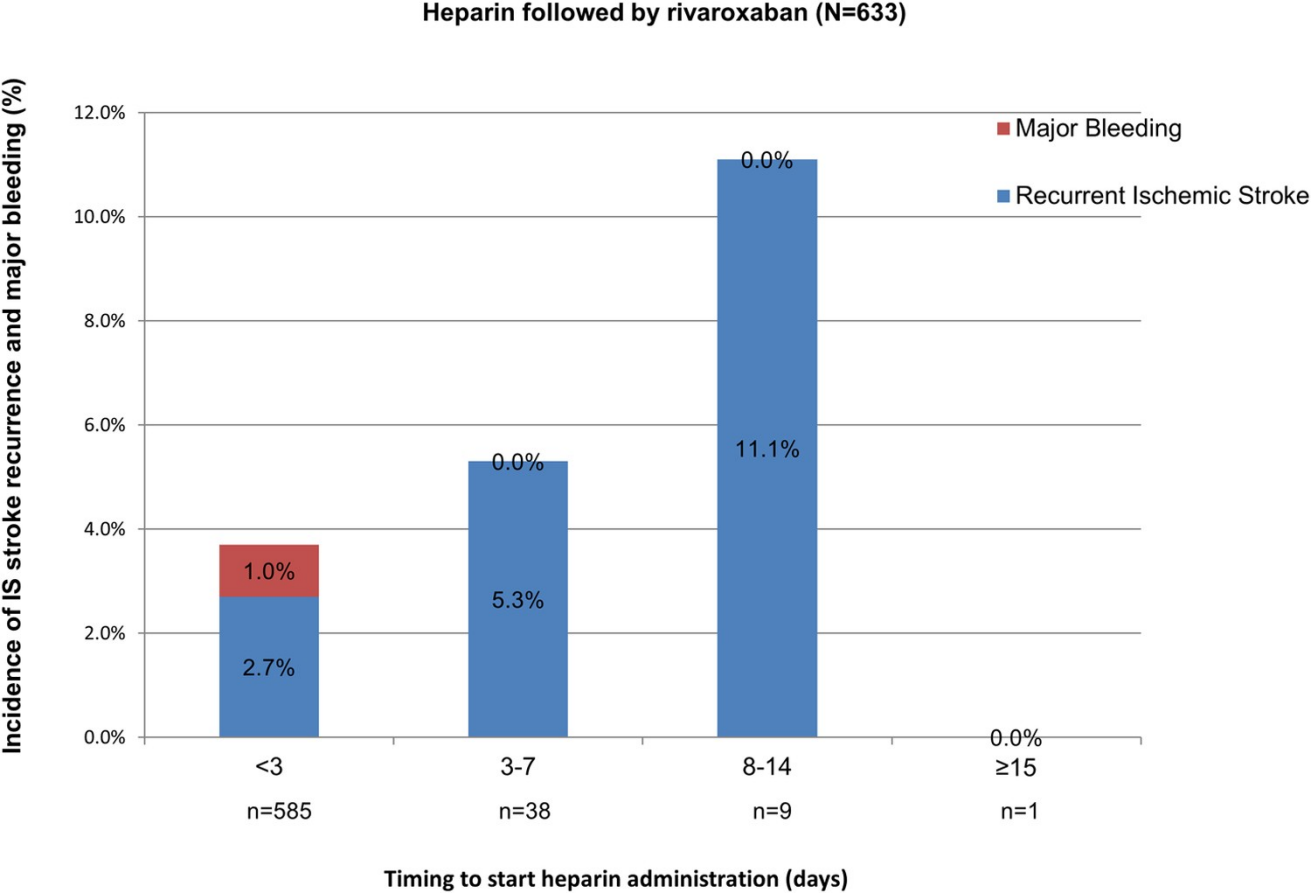
Recurrent ischemic stroke and major bleeding according to timing to start anticoagulation in patients treated with heparin followed by rivaroxaban. IS, ischemic stroke.

**Fig 8:**
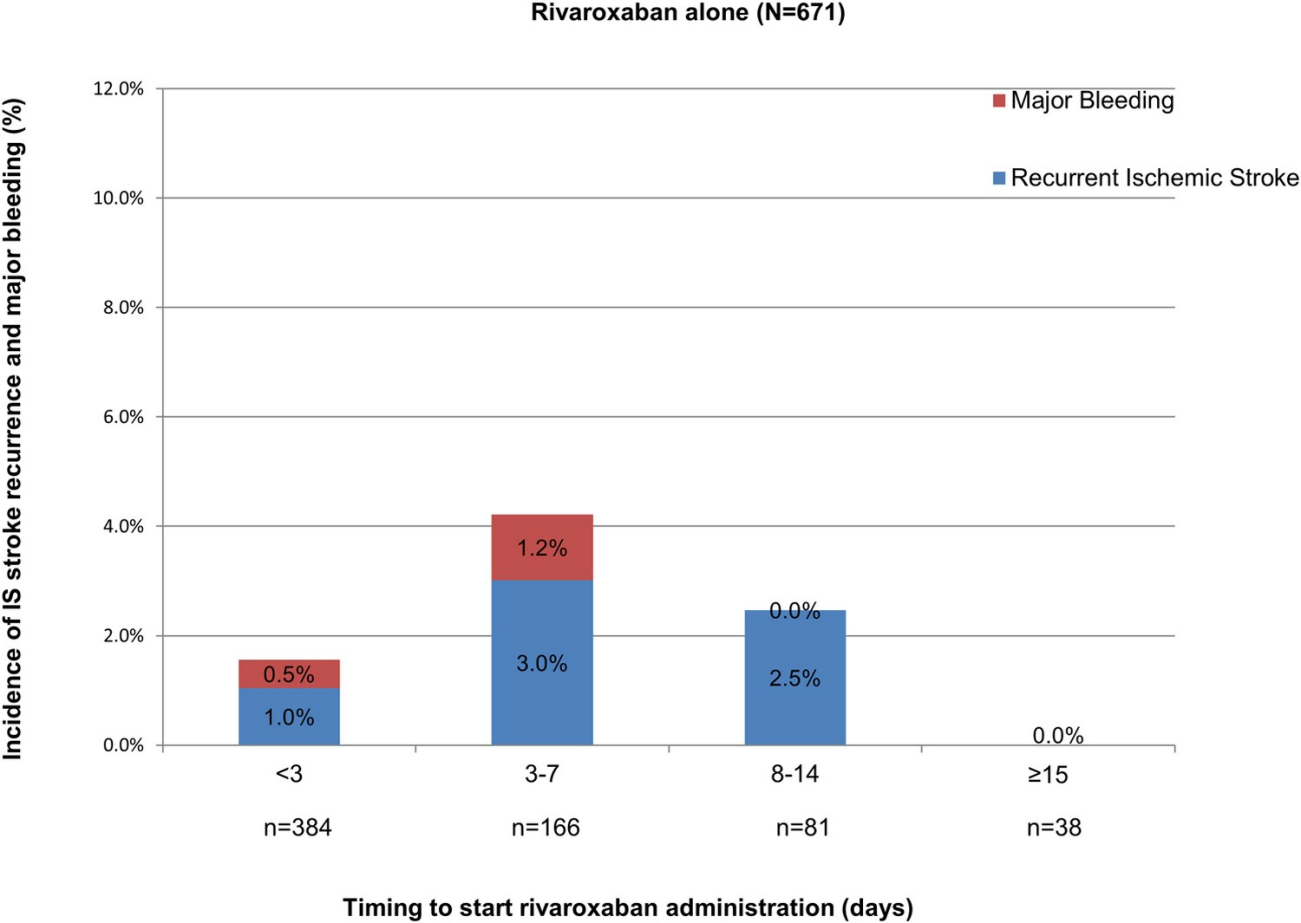
Recurrent ischemic stroke and major bleeding according to timing to start anticoagulation in patients treated with rivaroxaban alone. IS, ischemic stroke.

A median 10,000 U (10,000–12,000 U)/day of heparin followed by rivaroxaban was administered in 638 patients (heparin group, 48.8%) for a median of 3.3 days (1.8–6.3 days) and not in the other 669 (non-heparin group, two data were not available). The incidence of IS recurrence was higher among the heparin group (Fig 7) versus patients treated with rivaroxaban alone (Fig 8) (3.0% versus 1.6%, respectively. *P* = 0.1010); however, there was no significant difference. When we compared the incidence rates of IS recurrence or major bleeding events in the patients who started anticoagulation treatment <3 days after stroke onset, they were numerically higher in the heparin group than the non-heparin group (IS recurrence, 2.7% versus 1.0%, respectively, *P* = 0.104; major bleeding, 1.0% versus 0.5%, respectively, *P* = 1.0), but without significant difference.

## Discussion

This was a large-scale registration study with >1300 patients, in which we observed the usefulness of rivaroxaban for acute IS and TIA patients with NVAF. The key results of this study were that initiation of rivaroxaban administration in acute IS or TIA was associated with a low recurrence of IS (incidence of 2.3% [30/1309 patients]) and a low incidence of major bleeding events (0.8% [11/1309] patients), that recurrent IS occurred more frequently than major bleeding (2.3% and 0.8%, respectively. *P* = 0.0028, chi-square test), that the frequency of recurrent IS was significantly lower when rivaroxaban treatment started ≤14 days versus ≥15 days after the index IS/TIA (2.0% versus 6.8%, *P* = 0.0034, chi-square test, and confirmed by multivariate analysis, *P* = 0.0021), and that a small or intermediate cerebral infarct size and NIHSS score ≤3 were associated with an earlier start of rivaroxaban administration (median 2.9 days and median 2.8 days, respectively).

The low incidence of IS recurrence among those who initiated early rivaroxaban treatment in the present study was consistent with that reported by Seiffge et al [12]_._ In their study, NOACs were used for secondary prophylaxis in a sample of 204 patients with a similar mean age, atrial fibrillation (AF), and recent acute IS; they reported six events of IS recurrence among 204 patients (7.7%/y) [12]. A recently conducted study evaluating the current trends of OAC choice for acute stroke patients with NVAF in Japan reported that patients starting NOACs (dabigatran N = 203, rivaroxaban N = 238, apixaban N = 25) earlier had smaller infarcts and lower NIHSS scores [13]. The triple AXEL study, a randomized, multicenter, open-label trial, conducted in Korea, compared the efficacy and safety of acute anticoagulant therapy within 5 days of index IS between the rivaroxaban group (n = 95) and warfarin group (n = 88), with acute mild ischemic stroke associated with atrial fibrillation, and demonstrated that both treatments had comparable safety and efficacy [14]. In the rivaroxaban group, symptomatic recurrent ischemic stroke was seen only in one patient (1.1%) during an observational period of 4 weeks. It seems that the lower incidence of recurrent IS when rivaroxaban treatment started ≤14 days versus ≥15 days after the index IS/TIA in the present study supports current guidelines recommending that the optimal timing to start warfarin or NOACs is within 2 weeks of stroke onset [7,8].

The incidence of major bleeding and ICH in the present study was low. This was consistent with that reported in the study by Seiffge et al; however, in their study, the risk of ICH was low regardless of the timing of NOAC initiation [12]. Our results are also in line with those of a recent Japanese study assessing the 3-month risk-benefit profile of anticoagulation after stroke in patients with NVAF, in which the rates of major bleeding, particularly ICH, were lower with NOACs than with warfarin, although the differences were not significant [15]. The triple AXEL study also demonstrated low incidences of major bleeding (1.1%) and symptomatic ICH (0%) (n = 95) in the rivaroxaban group [14]. In Japan and other East Asian countries, the incidence of ICH is known to be markedly higher than that in Western countries [16,17]. However, several clinical trials of NOACs have shown a consistently and substantially lower incidence of ICH among Asians and non-Asians compared with those receiving warfarin [16,17]. As in previous studies, the low incidence of ICH observed in the present study may be attributed to the inhibitory coagulation mechanisms of NOACs on coagulation [16–18].

The RAF-NOACs study, which investigated early recurrence and major bleeding in 1,127 patients who had acute ischemic stroke associated with NVAF and started to take NOAC for the secondary prevention of stroke, demonstrated that a combined rate of ischemic embolic recurrence and severe bleeding was 5% within 90 days, as low as 3.1% (IS recurrence 2.3% and major bleeding 0.8%) of the current study, and a multivariate analysis suggested no significant marginal effect regarding the timing of administration despite the occurrences of the combined rate of ischemic embolic recurrence and severe bleeding having variability with respect to the timing of NOAC administration, 12.4% within 3 days, 2.1% between 3 and 14 days, and 9.1% beyond 14 days [19]. It included 366 patients who received rivaroxaban, in whom recurrent ischemic stroke was noted in only four patients (1.1%) and major bleeding including symptomatic hemorrhagic transformation was in only nine patients (2.5%). In the current study, recurrent IS occurred more frequently than major bleeding (2.3% and 0.8%, respectively. *P* = 0.0028), incidence of major bleeding was low (0.7%) when started within 3 days, and a small or intermediate cerebral infarct size was associated with an earlier start of rivaroxaban administration (median 2.9 days). Therefore, rivaroxaban started as early as 3 days or less after stroke onset may be a feasible treatment option for patients with a small or medium-sized infarction.

In the present study, multivariate analysis demonstrated that major bleeding was associated with use of antiplatelet therapy before onset. This is clinically relevant for the following reasons: first, aspirin and other antiplatelet therapies have irreversible effects on platelet aggregation, which may play a role in developing major bleeding early after the index IS/TIA. Second, major bleeding may be associated with vascular impairment frequently, which is seen in patients with several atherosclerotic diseases that require antiplatelet therapy.

Because the incidence of stroke recurrence was not higher among patients treated with rivaroxaban alone versus patients treated with heparin followed by rivaroxaban, it may be appropriate to initiate treatment with rivaroxaban monotherapy as an alternative to initial treatment with heparin. Heparin may be started early in patients without AF during hospitalization; however, it may be replaced with rivaroxaban once AF is observed. In the RAF-NOACs study, low molecular weight heparin (LMWH) was administered in 10% of patients before commencement of NOAC treatment [19]. The authors found that these patients had a significantly higher rate of major bleeding compared with those treated with NOAC alone and recommend not to start with LMWH before the commencement of NOACs.

The reason why the frequency of the rt-PA treatment was lower in patients starting rivaroxaban ≥15 days after stroke onset versus ≤14 days of stroke onset may reflect that rt-PA treatment can be difficult to perform and that treatment initiation with rivaroxaban can be delayed in the case of large infarctions.

### Limitations

This study has several limitations that may have led to biased results. The number of patients registered (1333) was lower than planned (2000), which may have led to underpowered results. The reasons for not meeting the target sample size as originally planned were that there were other treatment options available to patients at the time (three other NOACs and warfarin), promotion for study enrollment may have been inadequate, and we were unable to extend the enrollment period owing to limited research funding. When fitting Cox regression to the data, however, Firth’s penalized likelihood approach was used to cope with issues caused by the small number of events, and we consider that the issue of smaller sample size than that planned did not have a significant effect on our results and that the small occurrence rates of both ischemic stroke and major bleeding in the >1300 registered cases is an important finding that supports acute anticoagulant treatment with DOAC in acute ischemic stroke patients with NVAF. Because this was an observational study, not a randomized control trial, the timing to start rivaroxaban administration was determined by each investigator. Unfractionated heparin was used in approximately half of the patients; however, most patients were treated with low heparin doses (10,000 IU/day) during short periods. Because the power of a sub-analysis would be weak, the usefulness of heparin on the study treatment could not be judged from the present study; therefore, to clarify the appropriate timing to start rivaroxaban administration according to infarct size and the advantages and disadvantages of heparin administration, further prospective randomized trials are required. Of the total sample, 32.7% of patients underwent acute reperfusion therapy, endovascular treatment, or treatment with rt-PA, which depended on each investigator. Data on MRI-DWI were not available in 100 cases. Furthermore, the power of the subanalysis was decreased when the incidence of IS recurrence and major bleeding among patients treated with heparin followed by rivaroxaban was compared with those who received rivaroxaban monotherapy only. Finally, although the rivaroxaban dose (15/10 mg) approved in Japan differs from the dose approved in Western countries (20/15 mg), the pharmacokinetic and pharmacodynamic profiles of rivaroxaban 15/10 mg in Japanese patients were found to be similar to those of 20/15 mg in Caucasian populations [6,20]. J-ROCKET AF trial a prospective, randomized, double-blind phase III trial conducted in Japan confirmed non-inferiority of rivaroxaban (15/10 mg) to warfarin and supported bridging the global ROCIKET AF result into Japanese clinical practice [5, 6]. Thus, considering the smaller physique of Japanese subjects versus Caucasian patients, low-dose rivaroxaban (15/10 mg) seems more appropriate for Japanese versus Caucasian patients. Similarly, post-Marketing Alteplase Registration Study (J-MARS) demonstrated that thrombolysis with 0.6 mg/kg intravenous alteplase for acute ischemic stroke Japanese patients in routine clinical practice was comparable to that with 0.9 mg/kg alteplase used in North America and the European Union [21–23]. Therefore, the present results may be applicable to non-Japanese populations.

## Conclusions

Initiation of rivaroxaban administration in acute IS or TIA was associated with a low recurrence of IS (2.3%), and a low incidence of major bleeding events (0.8%) for 90 days after the index stroke. For the prevention of recurrent attack in acute IS patients with NVAF, we consider it feasible to start the administration of rivaroxaban within 14 days of onset. Rivaroxaban started within 3 days of stroke onset may also be a feasible treatment option for patients with a small or medium-sized infarction.

## Supporting information

S1 Appendix List of institutional review boards of participating study centers. (DOCX)

S1 Table Primary and secondary endpoint measures compared by timing to start rivaroxaban administration and size of infarct. (DOCX)

S2 Table. I ncidence of adverse events (AEs) (except hemorrhage) after starting rivaroxaban. (DOCX)
