# Supplementary material for: Rivaroxaban administration after acute ischemic stroke: The RELAXED study
Source: PLoS One. 2019 Feb 13;14(2):e0212354. doi: 10.1371/journal.pone.0212354 (PMC6373970; doi:10.1371/journal.pone.0212354)
Supplement: S1 Appendix — (DOCX) [file pone.0212354.s001.docx]

**S1 Appendix. List of Institutional Review Boards of participating study centers.**

National Hospital Organization Kyushu Medical center and National Cerebral and Cardiovascular Center and other ethics committees of Shinsapporo Neurosurgical Hospital, Hokuto Hospital, Otaru General Hospital, Japan Red Cross Hachinohe Hospital, Ohnishi Neurological Center, Japanese Red Cross Asahikawa Hospital, Tosei General Hospital, Koshigaya Municipal Hospital,Yokosuka Kyosai Hospital, Noshiro Kosei Medical Center, Nagatomi Neurosugical Hospital, Kohnan Hospital, Yamagata Prefectural Central Hospital, Shonan Kamakura General Hospital, Yokohama Shintoshi Neurosurgical Hospital, Nishinomiya Kyoritsu Neurosurgical Hospital, Chikamori Health Care Group, Chikamori Hospital, Teine Keijinkai Hospital, Kobe City Medical Center General Hospital, Local Incorporated Administrative Agency, Sasebo City General Hospital, Japanese Red Cross Kumamoto Hospital, Kochi Health Sciences Center, Showa University Koto Toyosu Hospital, Hakodate Shintoshi Hospital, Juntendo University Shizuoka Hospital, Muroran City General Hospital, Japanese Red Cross Fukui Hospital, National Hospital Organization Nagoya Medical Center, Kurashiki Central Hospital, Saitama Medical Center, Kyoto University Hospital, Kawasaki Medical School Hospital , Shiga University of Medical Science Hospital, Abashiri Neurosurgical and Rehabilitation Hospital, Chikamori Health Care Group, Chikamori Hospital, Doutou Neurosurgery, Tokyo Medical University Ibaraki Medical Center, Nanbu Medical Center / Nanbu Child Medical Center, Shiroyama Hospital, Hyogo College Of Medicine, Tokushima University Hospital, Japanese Red Cross Fukushima Hospital, Nagoya Ekisaikai Hospital, Saiseikai Fukuoka General Hospital, Saga University Hospital, Mihara Memorial Hospital, Fukuoka Tokushukai Hospital, Takagi Hospital, Sonoda Daiichi Hospital, Saiseikai Yokohama Tobu Hospital, Kawasaki Medical School Medical Center, St. Mary's Hospital, Toyota Memorial Hospital, Obihiro Kosei General Hospital, Hokkaido Neurosugical Memorial Hospital, Toranomon Hospital, The Jikei University Hospital, Higashiyamato Hospital, Saitama Medical University International Medical Center, Kumagaya General Hospital, Yokohama Rosai Hospital, Rinku General Medical Center, Yamaguchi Prefectural Grand Medical Center, Wakayama Medical University, National Cerebral and Cardiovascular Center, Nakamura Memorial Hospital, Ina Central Hospital, Japan Community Health Care Organization Chukyo Hospital, Osaka City General Hospital, National Hospital Organization Osaka National Hospital, Saitama Medical Center, National Hospital Organization Kagoshima Medical Center, Osaka Neurosurgical Hospital, Fukuoka University Hospital, Tokorozawa Central Hospital, Rapport Tanabe Neuro Hospital, Nippon Medical School Hospital, Tokyo Saiseikai Central Hospital, St. Mariannna University School of Medicine, Toyota Kosei Hospital, Junshin Hospital, Naha City Hospital, Juntendo University Hospital, Koseikai Takeda Hospital, Healthcare Corporation Suiseikai Kajikawa Hospital, Tokyo Women's Medical University Hospital, Iwate Medical University, Nihonkai General Hospital, Iwate Prefectural Kuji Hospital, Masu Hospital, Kuwana Hospital, Tsuchiura Kyodo General Hospital, Japanese Red Cross Maebashi Hospital, Yokohama Sakae Kyosai Hospital, Federation of National Public Service Personnel Mutual Associations, Seirei Mikatahara General Hospital, Kumamoto City Hospital, Japanese Red Cross Ise Hospital, St. Marianna University School of Medicine Toyoko Hospital, Sioda Hospital, Keio University Hospital, Tokai University Hospital, National Hospital Organization Kure Medical Center and Chugoku Cancer Center, Kokura Memorial Hospital, National Hospital Oraganization Maizuru Medical Center, Tsukuba Medical Center Hospital, Seirei Memorial Hospital, Toyama University Hospital, Japanese Red Cross Nagoya Daini Hospital, Tominaga Hospital, Nishiwaki Municipal Hospital, Okayama University Hospital, Hiroshima City Hiroshima Citizens Hospital, Steel Memorial Yawata Hospital, Hyogo Brain and Heart Center, Nagasaki Harbor Medical Center, Imamura Hospital, Kagoshima City Hospital, Toho Unversity Omori Medical Center, Fukuoka Wajiro Hospital, Nagano Matsushiro General Hospital, Japanese Red Cross Musashino Hospital, Nippon Medical School Ciba Hokusoh Hospital, Japanese Red Cross Shizuoka Hospital, Uji-Tokushukai Medical Center, Osaka University Hospital, Japan Community Health Care Organization Hoshigaoka Medical Center, Shin Koga Hospital, Fujimoto General Hospital, Saiseikai Nagasaki Hospital, HITO Medical Center, Niigata Prefectural Shibata Hospital, Ichikikushikino Medical Association standing Neurosurgery Center, Fukui Prefectural Hospital, Atsuji Neurosurgery Hospital, Shinko Hospital, Kenko-kai Sogo Tokyo Hospital, Tonami General Hospital, Hirosaki Stroke and Rehabilitation Center, Dokkyo Medical University, JA Toride Medical Center, Tachikawa Hospital, Kanazawa Neurosurgical Hospital, National Hospital Organization Yokohama Medical Center, Toyohashi Municipal Hospital, Japanese Red Cross Kyoto Daini Hospital

Osaka Neurological Institute, Tenri Hospital, Ehime Prefectural Central Hospital

Saiseikai Kumamoto Hospital, National Hospital Organization Kumamoto Medical Center, Japanese Red Cross Ashikaga Hospital, Osaka City University Graduate School of Medicine And Faculty of Medicine, Kyusyu Rosai Hospital, Japanese Red Cross Nagoya Daiichi Hospital, Saga-Ken Medical Cntre Koseikan.
