# Supplementary material for: Rivaroxaban administration after acute ischemic stroke: The RELAXED study
Source: PLoS One. 2019 Feb 13;14(2):e0212354. doi: 10.1371/journal.pone.0212354 (PMC6373970; doi:10.1371/journal.pone.0212354)
Supplement: S1 Table — (DOCX) [file pone.0212354.s002.docx]

**S1 Table. Primary and secondary endpoint measures compared by timing to start rivaroxaban administration and size of infarct.**

|  | Size of cerebral infarct | Timing to start rivaroxaban administration | | | | | |
| --- | --- | --- | --- | --- | --- | --- | --- |
|  |  | <3 days | 3–7 days | 8–14 days | ≥15 days | Unknown |  |
| Number | Total, 1309 | 584 | 435 | 198 | 88 | 4 |  |
|  | Small (<4.0 cm^3^), 411 | 213 | 138 | 48 | 11 | 1 |  |
|  | Medium (≥4.0 and <22.5 cm^3^), 394 | 199 | 122 | 53 | 19 | 1 |  |
|  | Large (≥22.5 cm^3^), 404 | 115 | 152 | 88 | 49 | 0 |  |
|  | Unknown, 100 | 57 | 23 | 9 | 9 | 2 |  |
| Event |  | n (%) | n (%) | n (%) | n (%) |  | *X^2^*-test  *P*-value |
| Recurrent IS | Total | 9 (1.5) | 11 (2.5) | 4 (2.0) | 6 (6.8) | 0 | 0.0216 |
|  | Small (<4.0 cm^3^) | 2 (0.9) | 2 (1.4) | 1 (2.1) | 1 (9.1) | 0 | 0.1738 |
|  | Medium (≥4.0 and <22.5 cm^3^) | 5 (2.5) | 5 (4.1) | 2 (3.8) | 0 | 0 | 0.7201 |
|  | Large (≥22.5 cm^3^) | 2 (1.7) | 3 (2.0) | 0 | 5 (10.2) | 0 (-) | 0.0020 |
|  | Unknown | 0 | 1 (4.3) | 1 (11.1) | 0 | 0 | 0.1276 |
| Major bleeding | Total | 4 (0.7) | 5 (1.1) | 2 (1.0) | 0 | 0 | 0.6874 |
|  | Small (<4.0 cm^3^) | 0 | 2 (1.4) | 0 | 0 | 0 | 0.2657 |
|  | Medium (≥4.0 and <22.5 cm^3^) | 3 (1.5) | 0 | 1 (1.9) | 0 | 0 | 0.5085 |
|  | Large (≥22.5 cm^3^) | 1 (0.9) | 3 (2.0) | 1 (1.1) | 0 | 0 (-) | 0.7002 |
|  | Unknown | 0 | 0 | 0 | 0 | 0 | - |
| Composite of IS and TIA | Total | 10 (1.7) | 13 (3.0) | 5 (2.5) | 7 (8.0) | 0 | 0.0087 |
|  | Small (<4.0 cm^3^) | 3 (1.4) | 3 (2.2) | 1 (2.1) | 1 (9.1) | 0 | 0.3478 |
|  | Medium (≥4.0 and <22.5 cm^3^) | 5 (2.5) | 5 (4.1) | 2 (3.8) | 1 (5.3) | 0 | 0.8267 |
|  | Large (≥22.5 cm^3^) | 2 (1.7) | 4 (2.6) | 1 (1.1) | 5 (10.2) | 0 (-) | 0.0142 |
|  | Unknown | 0 | 1 (4.3) | 1 (11.1) | 0 | 0 | 0.1276 |
| Composite cardiovascular events | Total | 21 (3.6)] | 21 (4.8) | 10 (5.1) | 11 (12.5) | 0 | 0.0042 |
|  | Small (<4.0 cm^3^) | 4 (1.9) | 4 (2.9) | 2 (4.2) | 2 (18.2) | 0 | 0.0178 |
|  | Medium (≥4.0 and <22.5 cm^3^) | 10 (5.0) | 8 (6.6) | 4 (7.5) | 2 (10.5) | 0 | 0.7319 |
|  | Large (≥22.5 cm^3^) | 7 (6.1) | 8 (5.3) | 2 (2.3) | 7 (14.3) | 0 (-) | 0.0392 |
|  | Unknown | 0 | 1 (4.3) | 2 (22.2) | 0 | 0 | 0.0039 |
| Any bleeding event | Total | 62 (10.6) | 82 (18.9) | 41 (20.7) | 36 (40.9) | 0 | <0.0001 |
|  | Small (<4.0 cm^3^) | 5 (2.3) | 3 (2.2) | 0 | 1 (9.1) | 0 | 0.3160 |
|  | Medium (≥4.0 and <22.5 cm^3^) | 31 (15.6) | 29 (23.8) | 14 (26.4) | 6 (31.6) | 0 | 0.0955 |
|  | Large (≥22.5 cm^3^) | 24 (20.9) | 48 (31.6) | 26 (29.5) | 28 (57.1) | 0 (-) | <0.0001 |
|  | Unknown | 2 (3.5) | 2 (8.7) | 1 (11.1) | 1 (11.1)] | 0 | 0.6321 |
| Intracranial hemorrhage | Total | 1 (0.2) | 4 (0.9) | 0 | 0 | 0 | 0.1670 |
|  | Small (<4.0 cm^3^) | 0 | 2 (1.4) | 0 | 0 | 0 | 0.2657 |
|  | Medium (≥4.0 and <22.5 cm^3^) | 1 (0.5) | 0 | 0 | 0 | 0 | 0.8067 |
|  | Large (≥22.5 cm^3^) | 0 | 2 (1.3) | 0 | 0 | 0 (-) | 0.3432 |
|  | Unknown | 0 | 0 | 0 | 0 | 0 | - |
| Hemorrhagic infarction | Total | 60 (10.3) | 77 (17.7) | 41 (20.7) | 36 (40.9) | 0 | <0.0001 |
|  | Small (<4.0 cm^3^) | 5 (2.3) | 1 (0.7) | 0 | 1 (9.1) | 0 | 0.1260 |
|  | Medium (≥4.0 and <22.5 cm^3^) | 30 (15.1) | 29 (23.8) | 14 (26.4)] | 6 (31.6) | 0 | 0.0711 |
|  | Large (≥22.5 cm^3^) | 23 (20.0) | 45 (29.6) | 26 (29.5) | 28 (57.1) | 0 (-) | <0.0001 |
|  | Unknown | 2 (3.5) | 2 (8.7) | 1 (11.1) | 1 (11.1) | 0 | 0.6321 |
| Adverse events | Total | 27 (4.6) | 28 (6.4) | 11 (5.6) | 6 (6.8) | 0 | 0.5962 |
|  | Small (<4.0 cm^3^) | 8 (3.8) | 7 (5.1) | 1 (2.1) | 1 (9.1) | 0 | 0.6662 |
|  | Medium (≥4.0 and <22.5 cm^3^) | 11 (5.5) | 7 (5.7) | 5 (9.4) | 0 | 0 | 0.4833 |
|  | Large (≥22.5 cm^3^) | 6 (5.2) | 14 (9.2) | 4 (4.5) | 5 (10.2) | 0 (-) | 0.3626 |
|  | Unknown | 2 (3.5) | 0 | 1 (11.1) | 0 | 0 | 0.3894 |

Abbreviations: IS, ischemic stroke; TIA, transient ischemic attack.
