# Supplementary material for: Rivaroxaban administration after acute ischemic stroke: The RELAXED study
Source: PLoS One. 2019 Feb 13;14(2):e0212354. doi: 10.1371/journal.pone.0212354 (PMC6373970; doi:10.1371/journal.pone.0212354)
Supplement: S2 Table. I — (DOCX) [file pone.0212354.s003.docx]

**S2 Table. Incidence of adverse events (AEs) (except hemorrhage) after starting rivaroxaban.**

| Adverse events | Total | | Timing to start rivaroxaban administration | | | | | | | | | |
| --- | --- | --- | --- | --- | --- | --- | --- | --- | --- | --- | --- | --- |
| System organ class  Preferred term |  |  | <3 days | | 3–7 days | | 8–14 days | | ≥15 days | | Unknown | |
| N | 1309 | | 584 | | 435 | | 198 | | 88 | | 4 | |
|  | n | (%) | n | (%) | n | (%) | n | (%) | n | (%) | n | (%) |
| Cardiac disorders | 21 | (1.6) | 13 | (2.2) | 5 | (1.1) | 2 | (1.0) | 1 | (1.1) | 0 | (0.0) |
| Cardiac failure | 8 | (0.6) | 4 | (0.7) | 2 | (0.5) | 2 | (1.0) | 0 | (0.0) | 0 | (0.0) |
| Cardiac failure acute | 2 | (0.2) | 1 | (0.2) | 0 | (0.0) | 0 | (0.0) | 1 | (1.1) | 0 | (0.0) |
| Cardiac failure congestive | 2 | (0.2) | 2 | (0.3) | 0 | (0.0) | 0 | (0.0) | 0 | (0.0) | 0 | (0.0) |
| Arrhythmia | 1 | (0.1) | 1 | (0.2) | 0 | (0.0) | 0 | (0.0) | 0 | (0.0) | 0 | (0.0) |
| Atrial fibrillation | 1 | (0.1) | 1 | (0.2) | 0 | (0.0) | 0 | (0.0) | 0 | (0.0) | 0 | (0.0) |
| Bradycardia | 1 | (0.1) | 1 | (0.2) | 0 | (0.0) | 0 | (0.0) | 0 | (0.0) | 0 | (0.0) |
| Tachycardia | 1 | (0.1) | 0 | (0.0) | 1 | (0.2) | 0 | (0.0) | 0 | (0.0) | 0 | (0.0) |
| Ventricular tachycardia | 1 | (0.1) | 1 | (0.2) | 0 | (0.0) | 0 | (0.0) | 0 | (0.0) | 0 | (0.0) |
| Intracardiac thrombus | 1 | (0.1) | 1 | (0.2) | 0 | (0.0) | 0 | (0.0) | 0 | (0.0) | 0 | (0.0) |
| Atrial thrombosis | 1 | (0.1) | 0 | (0.0) | 1 | (0.2) | 0 | (0.0) | 0 | (0.0) | 0 | (0.0) |
| Left ventricular dysfunction | 1 | (0.1) | 1 | (0.2) | 0 | (0.0) | 0 | (0.0) | 0 | (0.0) | 0 | (0.0) |
| Sinus node dysfunction | 1 | (0.1) | 0 | (0.0) | 1 | (0.2) | 0 | (0.0) | 0 | (0.0) | 0 | (0.0) |
| Infections and infestations | 16 | (1.2) | 3 | (0.5) | 9 | (2.1) | 2 | (1.0) | 2 | (2.3) | 0 | (0.0) |
| Pneumonia | 8 | (0.6) | 1 | (0.2) | 5 | (1.1) | 2 | (1.0) | 0 | (0.0) | 0 | (0.0) |
| Urinary tract infection | 3 | (0.2) | 2 | (0.3) | 1 | (0.2) | 0 | (0.0) | 0 | (0.0) | 0 | (0.0) |
| Pseudomembranous colitis | 2 | (0.2) | 0 | (0.0) | 1 | (0.2) | 0 | (0.0) | 1 | (1.1) | 0 | (0.0) |
| Pulmonary tuberculosis | 1 | (0.1) | 0 | (0.0) | 0 | (0.0) | 0 | (0.0) | 1 | (1.1) | 0 | (0.0) |
| Pyelonephritis | 1 | (0.1) | 0 | (0.0) | 1 | (0.2) | 0 | (0.0) | 0 | (0.0) | 0 | (0.0) |
| Staphylococcal sepsis | 1 | (0.1) | 0 | (0.0) | 1 | (0.2) | 0 | (0.0) | 0 | (0.0) | 0 | (0.0) |
| Respiratory, thoracic and mediastinal disorders | 12 | (0.9) | 3 | (0.5) | 4 | (0.9) | 1 | (0.5) | 4 | (4.5) | 0 | (0.0) |
| Pneumonia aspiration | 9 | (0.7) | 3 | (0.5) | 2 | (0.5) | 0 | (0.0) | 4 | (4.5) | 0 | (0.0) |
| Aspiration | 1 | (0.1) | 0 | (0.0) | 0 | (0.0) | 1 | (0.5) | 0 | (0.0) | 0 | (0.0) |
| Interstitial lung disease | 1 | (0.1) | 0 | (0.0) | 1 | (0.2) | 0 | (0.0) | 0 | (0.0) | 0 | (0.0) |
| Pulmonary edema | 1 | (0.1) | 0 | (0.0) | 1 | (0.2) | 0 | (0.0) | 0 | (0.0) | 0 | (0.0) |
| Injury, poisoning and procedural complications | 6 | (0.5) | 3 | (0.5) | 2 | (0.5) | 1 | (0.5) | 0 | (0.0) | 0 | (0.0) |
| Brain herniation | 1 | (0.1) | 1 | (0.2) | 0 | (0.0) | 0 | (0.0) | 0 | (0.0) | 0 | (0.0) |
| Head injury | 1 | (0.1) | 0 | (0.0) | 0 | (0.0) | 1 | (0.5) | 0 | (0.0) | 0 | (0.0) |
| Vascular pseudoaneurysm | 1 | (0.1) | 0 | (0.0) | 1 | (0.2) | 0 | (0.0) | 0 | (0.0) | 0 | (0.0) |
| Muscle strain | 1 | (0.1) | 1 | (0.2) | 0 | (0.0) | 0 | (0.0) | 0 | (0.0) | 0 | (0.0) |
| Contusion | 1 | (0.1) | 1 | (0.2) | 0 | (0.0) | 0 | (0.0) | 0 | (0.0) | 0 | (0.0) |
| Lower limb fracture | 1 | (0.1) | 0 | (0.0) | 1 | (0.2) | 0 | (0.0) | 0 | (0.0) | 0 | (0.0) |
| Nervous system disorders | 6 | (0.5) | 3 | (0.5) | 2 | (0.5) | 1 | (0.5) | 0 | (0.0) | 0 | (0.0) |
| Dizziness | 1 | (0.1) | 0 | (0.0) | 1 | (0.2) | 0 | (0.0) | 0 | (0.0) | 0 | (0.0) |
| Epilepsy | 1 | (0.1) | 0 | (0.0) | 0 | (0.0) | 1 | (0.5) | 0 | (0.0) | 0 | (0.0) |
| Headache | 1 | (0.1) | 0 | (0.0) | 1 | (0.2) | 0 | (0.0) | 0 | (0.0) | 0 | (0.0) |
| Hypoaesthesia | 1 | (0.1) | 0 | (0.0) | 1 | (0.2) | 0 | (0.0) | 0 | (0.0) | 0 | (0.0) |
| Myelopathy | 1 | (0.1) | 1 | (0.2) | 0 | (0.0) | 0 | (0.0) | 0 | (0.0) | 0 | (0.0) |
| Sensory disturbance | 1 | (0.1) | 1 | (0.2) | 0 | (0.0) | 0 | (0.0) | 0 | (0.0) | 0 | (0.0) |
| Carotid artery occlusion | 1 | (0.1) | 1 | (0.2) | 0 | (0.0) | 0 | (0.0) | 0 | (0.0) | 0 | (0.0) |
| Renal and urinary disorders | 6 | (0.5) | 3 | (0.5) | 3 | (0.7) | 0 | (0.0) | 0 | (0.0) | 0 | (0.0) |
| Renal impairment | 4 | (0.3) | 2 | (0.3) | 2 | (0.5) | 0 | (0.0) | 0 | (0.0) | 0 | (0.0) |
| Neurogenic bladder | 1 | (0.1) | 0 | (0.0) | 1 | (0.2) | 0 | (0.0) | 0 | (0.0) | 0 | (0.0) |
| Renal disorder | 1 | (0.1) | 1 | (0.2) | 0 | (0.0) | 0 | (0.0) | 0 | (0.0) | 0 | (0.0) |
| Renal failure | 1 | (0.1) | 0 | (0.0) | 1 | (0.2) | 0 | (0.0) | 0 | (0.0) | 0 | (0.0) |
| Hepatobiliary disorders | 4 | (0.3) | 1 | (0.2) | 2 | (0.5) | 1 | (0.5) | 0 | (0.0) | 0 | (0.0) |
| Hepatic function abnormal | 4 | (0.3) | 1 | (0.2) | 2 | (0.5) | 1 | (0.5) | 0 | (0.0) | 0 | (0.0) |
| Gastrointestinal disorders | 3 | (0.2) | 1 | (0.2) | 2 | (0.5) | 0 | (0.0) | 0 | (0.0) | 0 | (0.0) |
| Chronic gastritis | 1 | (0.1) | 1 | (0.2) | 0 | (0.0) | 0 | (0.0) | 0 | (0.0) | 0 | (0.0) |
| Dysphagia | 1 | (0.1) | 0 | (0.0) | 1 | (0.2) | 0 | (0.0) | 0 | (0.0) | 0 | (0.0) |
| Inguinal hernia | 1 | (0.1) | 0 | (0.0) | 1 | (0.2) | 0 | (0.0) | 0 | (0.0) | 0 | (0.0) |
| Investigations | 3 | (0.2) | 0 | (0.0) | 2 | (0.5) | 1 | (0.5) | 0 | (0.0) | 0 | (0.0) |
| Prothrombin time prolonged | 2 | (0.2) | 0 | (0.0) | 1 | (0.2) | 1 | (0.5) | 0 | (0.0) | 0 | (0.0) |
| Blood pressure decreased | 1 | (0.1) | 0 | (0.0) | 1 | (0.2) | 0 | (0.0) | 0 | (0.0) | 0 | (0.0) |
| Musculoskeletal and connective tissue disorders | 2 | (0.2) | 0 | (0.0) | 1 | (0.2) | 1 | (0.5) | 0 | (0.0) | 0 | (0.0) |
| Chondrocalcinosis pyrophosphate | 2 | (0.2) | 0 | (0.0) | 1 | (0.2) | 1 | (0.5) | 0 | (0.0) | 0 | (0.0) |
| Neoplasms benign, malignant and unspecified | 2 | (0.2) | 1 | (0.2) | 1 | (0.2) | 0 | (0.0) | 0 | (0.0) | 0 | (0.0) |
| Lung neoplasm malignant | 1 | (0.1) | 0 | (0.0) | 1 | (0.2) | 0 | (0.0) | 0 | (0.0) | 0 | (0.0) |
| Gingival cancer | 1 | (0.1) | 1 | (0.2) | 0 | (0.0) | 0 | (0.0) | 0 | (0.0) | 0 | (0.0) |
| Psychiatric disorders | 2 | (0.2) | 1 | (0.2) | 0 | (0.0) | 0 | (0.0) | 1 | (1.1) | 0 | (0.0) |
| Depression | 1 | (0.1) | 0 | (0.0) | 0 | (0.0) | 0 | (0.0) | 1 | (1.1) | 0 | (0.0) |
| Hallucination | 1 | (0.1) | 1 | (0.2) | 0 | (0.0) | 0 | (0.0) | 0 | (0.0) | 0 | (0.0) |
| Blood and lymphatic system disorders | 1 | (0.1) | 0 | (0.0) | 1 | (0.2) | 0 | (0.0) | 0 | (0.0) | 0 | (0.0) |
| Anaemia | 1 | (0.1) | 0 | (0.0) | 1 | (0.2) | 0 | (0.0) | 0 | (0.0) | 0 | (0.0) |
| Endocrine disorders | 1 | (0.1) | 0 | (0.0) | 1 | (0.2) | 0 | (0.0) | 0 | (0.0) | 0 | (0.0) |
| Thyroiditis | 1 | (0.1) | 0 | (0.0) | 1 | (0.2) | 0 | (0.0) | 0 | (0.0) | 0 | (0.0) |
| Metabolism and nutrition disorders | 1 | (0.1) | 0 | (0.0) | 0 | (0.0) | 1 | (0.5) | 0 | (0.0) | 0 | (0.0) |
| Gout | 1 | (0.1) | 0 | (0.0) | 0 | (0.0) | 1 | (0.5) | 0 | (0.0) | 0 | (0.0) |
| Skin and subcutaneous tissue disorders | 1 | (0.1) | 0 | (0.0) | 0 | (0.0) | 1 | (0.5) | 0 | (0.0) | 0 | (0.0) |
| Drug eruption | 1 | (0.1) | 0 | (0.0) | 0 | (0.0) | 1 | (0.5) | 0 | (0.0) | 0 | (0.0) |
| Vascular disorders | 1 | (0.1) | 1 | (0.2) | 0 | (0.0) | 0 | (0.0) | 0 | (0.0) | 0 | (0.0) |
| Arteriovenous fistula | 1 | (0.1) | 1 | (0.2) | 0 | (0.0) | 0 | (0.0) | 0 | (0.0) | 0 | (0.0) |
